# Association of the Intestinal Microbiome with the Development of Neovascular Age-Related Macular Degeneration

**DOI:** 10.1038/srep40826

**Published:** 2017-01-17

**Authors:** Martin S. Zinkernagel, Denise C. Zysset-Burri, Irene Keller, Lieselotte E. Berger, Alexander B. Leichtle, Carlo R. Largiadèr, Georg M. Fiedler, Sebastian Wolf

**Affiliations:** 1 Department of Ophthalmology, Inselspital, Bern University Hospital, University of Bern, Bern, Switzerland; 2 Department of Clinical Research, University of Bern, Bern, Switzerland; 3 Swiss Institute of Bioinformatics, Bern, Switzerland; 4 University Insitute of Clinical Chemistry, Inselspital, Bern University Hospital, University of Bern, Bern, Switzerland

## Abstract

Age-related macular degeneration (AMD) is the most frequent cause of blindness in the elderly. There is evidence that nutrition, inflammation and genetic risk factors play an important role in the development of AMD. Recent studies suggest that the composition of the intestinal microbiome is associated with metabolic diseases through modulation of inflammation and host metabolism. To investigate whether compositional and functional alterations of the intestinal microbiome are associated with AMD, we sequenced the gut metagenomes of patients with AMD and controls. The genera *Anaerotruncus* and *Oscillibacter* as well as *Ruminococcus torques* and *Eubacterium ventriosum* were relatively enriched in patients with AMD, whereas *Bacteroides eggerthii* was enriched in controls. Patient’s intestinal microbiomes were enriched in genes of the L-alanine fermentation, glutamate degradation and arginine biosynthesis pathways and decreased in genes of the fatty acid elongation pathway. These findings suggest that modifications in the intestinal microbiome are associated with AMD, inferring that this common sight threatening disease may be targeted by microbiome-altering interventions.

Age-related macular degeneration (AMD; Fig. 1a) is the leading cause of severe visual impairment in the elderly population of industrialized countries. AMD has become a significant global health issue^1^, and the prevalence of AMD is projected to grow by 50 percent in the next decades^2^. It is a complex disease that is caused by a combination of environmental and genetic factors. Several studies have shown that diet is a major modifiable risk factor for AMD development^3^,^4^,^5^,^6^,^7^,^8^. Intestinal microbial organisms and their genomes, collectively termed microbiome, play a major role in the digestion of food and in this way influence the global metabolism of their host^9^. The intestinal microbiome forms a complex ecosystem of up to 100 trillion microbes and shows substantial diversity of gut communities among individuals^10^. However, changes in microbiome composition can potentially modulate or alternatively stress host metabolism and may act as sources of inflammation and disease^11^. Recent findings have identified the intestinal microbiome as a contributor to metabolic diseases such as atherosclerosis, which is associated with lipid accumulation and inflammation in the arterial wall^12^. Furthermore, a recent report has suggested that the intestinal microbiome may trigger autoimmune response in the eye through activation signals to retina specific T cells^13^. Given the link between AMD and diet, the composition of the intestinal microbiome may also influence AMD development and progression. This link has recently been substantiated in an animal model, where alterations in the intestinal microbiome have resulted in exacerabation of choroidal neovascularization, a hallmark of neovascular AMD^14^. Neovascular AMD is caused by choroidal neovascularization, which often results in exudation and hemorrhage, leading to photoreceptor damage and central visual loss.

To address this issue, we investigated whether compositional and functional diversity of the intestinal microbiome is associated with AMD development and progression. To this end, we sequenced the intestinal metagenomes of patients with recent onset neovascular AMD and age- and sex-matched controls without AMD. For profiling microbial communities, we used the Metagenomic Phylogenetic Analysis (MetaPhlAn) tool^15^ and to infer the metabolic potential of the identified microbes, the HMP (Human Microbiome Project^16^) Unified Metabolic Analysis Network (HUMAnN)^17^ was applied. The data revealed an enrichment of *Oscillibacter, Anaerotruncus, Eubacterium ventriosum* and *Ruminococcus torques* in AMD patients versus *Bacteroides eggerthii* in controls. Our findings may have important implications for the prevention of this sight threatening disease as the intestinal microbiome may be modified by probiotics and antibiotics.

## Results

### Taxonomic characterization of the intestinal microbiome

To address whether the intestinal microbiome is associated with neovascular AMD, we sequenced the gut metagenomes of 12 patients with recent onset signs of neovascularization due to AMD and 11 age- and sex-matched controls without any signs of AMD (Table 1). In total, we generated 557 million 100 bp paired-end reads with an average insert size of 350 bp (on average, 24 ± 0.1 (s.d.) million reads per sample).

After trimming and filtering (see Methods), we obtained about 20 million non-human high-quality reads per sample for further analysis. On average, 4.6 ± 1.2% (s.d.) of the reads of a sample could be aligned to any marker sequence. This is by a factor of ten less than described in previous metagenomic studies using Illumina reads^12^,^18^. This discrepancy is due to the fact that MetaPhlAn maps reads against a catalogue of marker sequences including about 4% of the sequenced microbial genomes^15^. The majority of mapped reads were bacterial (99.2 ± 1.6% in patients and 99.7 ± 0.5% (s.d.) in controls). The microbiome composition was dominated by the phyla Bacteriodetes (65.3 ± 16.5% in AMD and 74.7 ± 12.6% (s.d.) in controls) and Firmicutes (29.0 ± 14.4% in AMD and 20.4 ± 8.9% (s.d.) in controls), followed by Actinobacteria (2.1 ± 3.2% in AMD and 1.6 ± 3.6% (s.d.) in controls) and Proteobacteria (1.6 ± 1.7% in AMD and 2.4 ± 3.0% (s.d.) in controls). Bacteroidia (65.3 ± 16.5% in AMD and 74.7 ± 12.6% (s.d.) in controls) and Clostridia (27.1 ± 13.4% in AMD and 19.4 ± 8.8% (s.d.) in controls) were shown to be the most abundant classes in our cohort (Fig. 2). This composition is consistent with previous observations^12^,^19^,^20^. The genera *Bacteroides* (36.5 ± 14.6% in AMD and 40.8 ± 23.1% (s.d.) in controls) and *Alistipes* (12.6 ± 6.4% in AMD and 13.8 ± 11.9% (s.d.) in controls) dominated the microbiome, followed by *Eubacterium* with a high intersubject variation (8.9 ± 8.6% in AMD and 5.3 ± 6.9% (s.d.) in controls). A total of 12 species were detected in all 23 samples, making up the core microbiome in our cohort, in agreement with previous observations^18^. *Bacteroides uniformis* was the most abundant species with 8.7 ± 6.0% in AMD and 6.1 ± 5.7% (s.d.) in controls, followed by *Alistipes onderdonkii* (4.4 ± 4.7% in AMD and 7.4 ± 10.5% (s.d.) in controls), *Subdoligranulum species* (4.8 ± 3.9% in AMD and 4.4 ± 6.1% (s.d.) in controls) and *Alistipes putredinis* (4.8 ± 4.6% in AMD and 4.1 ± 3.9% (s.d.) in controls).

### Distinct microbial composition

A principal component analysis with the health status as grouping variable revealed that differences in abundance of microbial species separated the patient group from the control group (Fig. 3a). To identify the taxa differing in relative abundance between AMD and controls, LEfSe was applied on the previously identified microbiome. The genera *Anaerotruncus* and *Oscillibacter* (and the family Oscillospiraceae) as well as *Ruminococcus torques* and *Eubacterium ventriosum* were enriched in AMD, whereas *Bacteroides eggerthii* was enriched in controls (p < 0.05, Kruksal-Wallis test; Fig. 3b,c).

### Metabolic functions of altered intestinal microbiome

To describe the metabolic features of the microbiome, we applied HUMAnN on each sample separately based on the taxonomic profiles from MetaPhlAn. Resulting organism-specific gene hits were functionally assigned to pathways using MinPath and their relative abundances were assessed. From the totally 2193 assigned organism-specific pathways, 253 pathways were detected in all samples making up the core metabolic functions in our cohort. A principal component analysis with the health status as grouping variable revealed that the difference in abundance of metabolic pathways separated the patient group from the control group (Fig. 4a). Applying LEfSe, 20 abundant pathways (i.e. occurring in at least 11 samples) were identified that differ between AMD patients and controls (Fig. 4b). Based on the distribution of pathway abundances in each sample with empiric p values > 0.05, we identified four differentially abundant pathways (Fig. 4c) using an empiric null distribution. L-alanine fermentation (GO 0019652), glutamate degradation (GO 0019671) and arginine biosynthesis (GO 0006525) pathways were up-regulated in the intestinal microbiome of AMD patients, whereas fatty acid elongation pathway (GO 00062) was enriched in the microbiome of controls (see Supplementary Fig. S1).

## Discussion

In this study, we identified several compositional and functional variations of the intestinal microbiome that may be related to the development of neovascular AMD. There is significant geographic and ethnic variability in the prevalence of AMD^2^. Although genetic susceptibility has been shown to play a substantial role in the pathogenesis of AMD and may contribute to the ethnic differences in its prevalence^21^, this variability may be further influenced by differences in the intestinal microbiome and diet, respectively. The intestinal microbiome consists of a complex community of microbes that impact normal physiology and susceptibility to disease mediated by inflammatory molecules such as lipopolysaccharides and peptidoglycans, which may contribute to metabolic disease^22^,^23^. The intestinal microbiome landscape seems to be fairly distinct and temporally stable in each individual. This suggests that a stable equilibrium exists for the microbiome. Furthermore, twins have more comparable microbiome compositions than unrelated individuals, suggesting that genetics may influence the microbiome composition. On the other hand, environmental factors may have a similar impact on microbiome composition, as monozygotic and dizygotic adult twins have equally similar microbiomes^11^. Recent studies have shown that the European microbiome is dominated by the *Bacteroides* enterotype, whereas the African microbiome was dominated by the *Prevotella* enterotype^24^.

We observed associations between neovascular AMD and microbiome composition at the taxonomical levels of bacterial genera and species. Furthermore, principal component analysis (PCA) on microbial species abundance confirmed a separation of patients and controls by microbiome composition (Fig. 3a). First, the relative abundance of *Bacteroides eggerthii* was increased in healthy age- and sex-matched controls. *Bacteroides* genera have been shown to play a major role in carbohydrate fermentation resulting in a pool of volatile fatty acids that are reabsorbed through the intestinal mucosa, providing a significant source of energy to the host^25^. *Bacteroides* can produce polysaccharide A (PSA), which may be involved in ligand-receptor interaction, modulating the immune response to pathogens. It has been indeed shown that PSA derived from *Bacteroides spp*. protect from experimental autoimmune encephalitis^26^. Secondly, the relative abundances of species such as *Ruminococcus torques*, a gram positive bacterium with mucin degradation capacities and *Oscillibacter*, which has been associated with high fat diet^27^, were increased in AMD patients. An increased *Oscillibacter* population has been shown to result in an increased gut permeability presumably mediated by a reduction in the mRNA expression tight junctions including ZO-1^28^. An increased relative abundance of *Anaerotruncus species* has been associated with aging and age-associated inflammation with elevated pro-inflammatory chemokines in a mouse model^29^. A similar finding has been reported for *Eubacterium ventriosum spp*. in humans which has been associated with elevated IL-6 and IL-8 cytokine levels^30^.

High-fat diets have been shown to exacerbate choroidal neovascularization in a mouse model by increasing the relative abundance of Firmicutes. These findings were associated with heightened intestinal permeability and chronic low-grade inflammation with elevated production of IL-6, IL-1b, TNF-a, and VEGF-A, cytokines that have been associated with the development of neovascular AMD^14^. A similar shift of relative abundance in Firmicutes at the expense of Bacteroidetes was observed in our cohort, with a relative abundance of Firmicutes of 29.0% in AMD and 20.4% in the control group.

Functional annotation analyses indicated that specific genes involved in individual metabolic pathways are enriched or decreased in patients with neovascular AMD. Although these genes allow to identify associated pathways and therefore can predict functional capabilities, quantification of messenger RNA and metabolic profiling to confirm functional differences are needed to further confirm these associations.

However, PCA on gene and pathway abundance revealed a separation of patients and controls by functional features (Fig. 4a). Our data revealed a decline in bacteria responsible for fatty acid elongation and an increase in L-alanine fermentation, glutamate degradation and arginine biosynthesis in patients with neovascular AMD. In the last decade, it has become evident that nutritional factors may influence the progression of AMD. A recent study has observed a decrease of some n-3 very long chain polyunsaturated fatty acids in the retina of eyes with early and intermediate AMD^31^. As such bioavailability of long chain polyunsaturated fatty acids is likely to have an impact on retinal physiology and may contribute to AMD. Glutamate is the main excitatory neurotransmitter in the retina and decreased availability of glutamate has been shown to result in deficient neurotransmission in the retina^32^. Furthermore, in patients with reduced ornithin aminotransferase activity increased levels of arginine are associated with progressive chorioretinal atrophy, suggesting an important role for arginine in the development of retinal degeneration^33^.

The best-validated supplementation therapy for AMD is the age-related eye disease study (AREDS) formulation, which contains antioxidant vitamin C and vitamin E, beta-carotene, copper and zinc. This combination has been shown to reduce risk toward progression of advanced AMD^34^. The bioavailability of several of these components may be influenced by the microbiome composition. This is supported by the large effect of the microbiome on mammalian plasma biochemistry which has been demonstrated by comparing plasma extracts from germ-free mice with samples from conventional mice^35^. Importantly, generation of certain antioxidants such as indole-3-propionic acid was shown to be completely dependent on the presence of intestinal microflora. Furthermore, it has been suggested that zinc absorption may be influenced by zinc competition between microbiome in the gastrointestinal tract of a host^36^.

This study cannot provide evidence for direct causal effects of an altered intestinal microbiome on the development of neovascular AMD. Study limitations are the small sample size which may not represent the entire AMD population, and furthermore, there are possible confounders due to genetic background and nutritional habit of patients and controls. However, if a specific taxonomic group or a metagenomic cluster annotated to a specific metabolic pathway is ultimately shown to be causally related to neovascular AMD, then long-term dietary interventions may allow modulation of an individual’s microbiome to avoid disease. The interplay between microbiome and the innate immune system, e.g. the complement system may provide a link between host genome and microbiome and remains an area of active investigation.

Our study suggests that the intestinal microbiome may play an important role in the development of neovascular AMD and may constitute a link between nutrition and development of this common disease.

## Methods

### Study design and recruitment

Participants (n = 23) were recruited from the Department of Ophthalmology of the University Hospital Bern (Inselspital), Switzerland. This study was conducted in accordance with the Declaration of Helsinki and approved by the Ethics Committee of the Canton of Bern (ClinicalTrials.gov: NCT02438111). After receiving oral and written information, all participants gave written informed consent to participate in the study. All participants were subjected to an ophthalmic examination including standard fundus color photography and optical coherence tomography. Patients (n = 12) had clinically confirmed recent onset of neovascular AMD and were 50 years of age or older. The control group (n = 11) was selected to represent an age- and sex-matched group with no signs of AMD. We tested for differences in a range of demographic values between the two groups using either Welch’s t test (for age and BMI) or Fisher’s exact test (for gender and smoking) in R v.3.2.1^37^. Further exclusion criteria for both groups were problems with the bowel movement, chronic inflammatory and gastrointestinal disease (including previous surgery in the gastrointestinal tract), recent history of use of systemic antibiotics within the last three months and opacities of ocular media.

### Sequencing and data quality control

Stool samples were brought chilled to the study center where they were immediately processed and frozen at −20 °C. Metagenomic DNA was isolated from 0.25 g of stool sample using the PowerSoil® DNA Isolation kit (Mo Bio Laboratories, Carlsbad, USA) according to the manufacturer’s protocol. The DNA was sent to BGI Europe (Copenhagen N, Denmark) for paired-end shotgun sequencing with up to ten samples pooled in one lane using the Illumina HiSeq 2000 platform. For library preparation, purified DNA was sheared by nebulization. The resulting overhangs were converted into blunt ends using T4 DNA polymerase, Klenow Fragment and T4 Polynucleotide kinase. After adding an ‘A’ base to the 3′ end of the blunt phosphorylated DNA fragments, adapter ligation was performed. Too short fragments were removed with Ampure beads and the resulting library was used for sequencing following standard pipelines of the Illumina platform, generating 100 bp paired-end reads. The reads were quality filtered with Trimmomatic v.0.32 as follows^38^: Illumina adapter sequences were removed and read ends were clipped off if the average base quality in a 4 bp window slid along the length of the read dropped below 15. We retained only reads of at least 80 bp after trimming. To remove sequences of human origin, all reads were mapped to the human reference genome hg19 using Bowtie2 v.2.2.4^39^ and only the unmapped reads were used for further analysis.

### Taxonomical analysis

**Figure 1:**
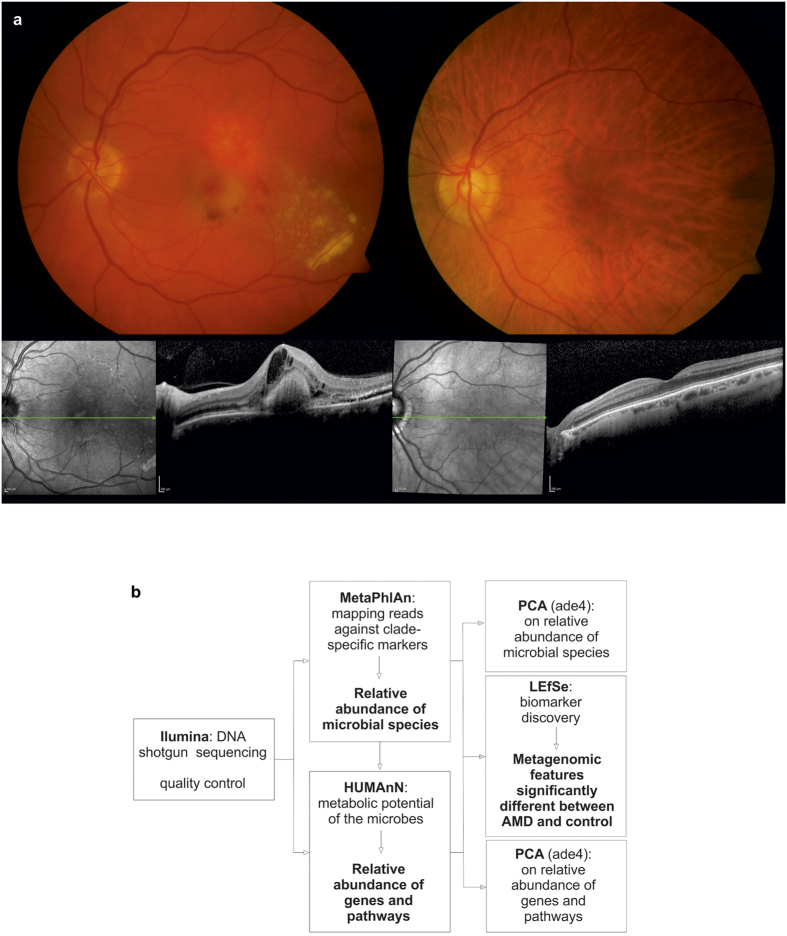
Disease phenotype and bioinformatics pipeline. (**a**) Representative color photographs and optical coherence tomography (OCT) images of a patient with neovascular age-related macular degeneration (AMD; left) and an age-matched control (right). (**b**) Illustration of our bioinformatics pipeline for analysing metagenomic data and its relation to neovascular AMD. Sequencing reads from stool probes were generated with high throughput sequencing and subjected to quality control. Species abundances were estimated by alignment of high quality reads to clade-specific marker sequences using the Metagenomic Phylogenetic Analysis (MetaPhlAn) tool. To describe the functional profile of the intestinal microbiome, the HMP (Human Microbiome Project) Unified Metabolic Analysis Network (HUMAnN) was used. The linear discriminant analysis (LDA) effect size (LEfSe) algorithm was applied to identify features significantly different between patients and controls. To provide a global analysis of microbial species and pathway abundance principal component analysis (PCA) using ade4 were performed.

**Figure 2:**
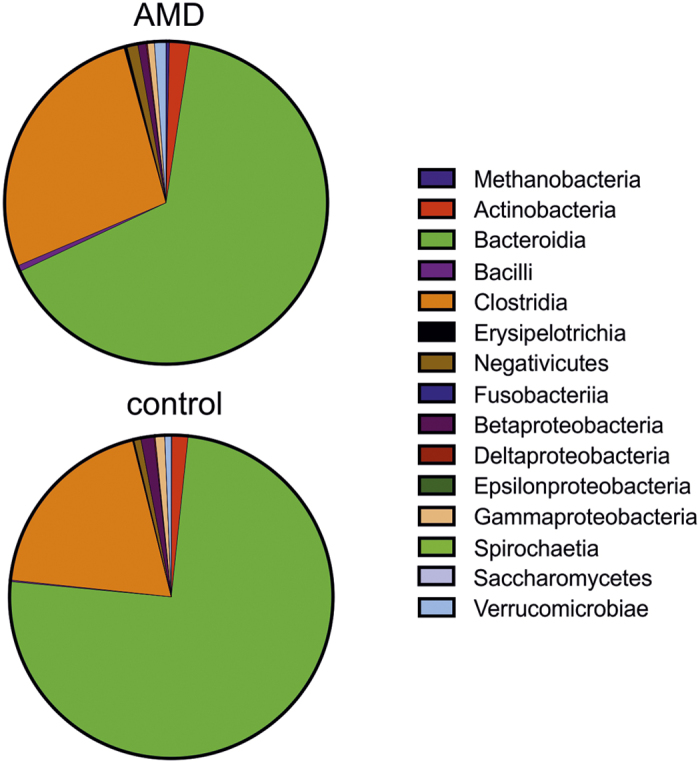
Taxonomic characterization of the intestinal microbiome using MetaPhlAn. Mean relative abundance of the major microbial classes among individuals of the cohort. Red are patients (age-related macular degeneration (AMD), n = 12), green are controls (n = 11).

The bioinformatics pipeline used for analysing the metagenomic data is illustrated in Fig. 1b. For microbial profiling, the high-quality non-human reads were mapped against a set of clade-specific markers (spanning bacteria and archaea at the species level or higher) using the Metagenomic Phylogenetic Analysis (MetaPhlAn) tool v.2.0 and the marker database v.20^15^ using default settings. In order to provide the relative abundance of each taxonomic unit, Bowtie2 v.2.2.4 was used for alignment (option “very-sensitive”) followed by normalization of the total number of reads in each clade by the nucleotide length of its marker.

**Figure 3:**
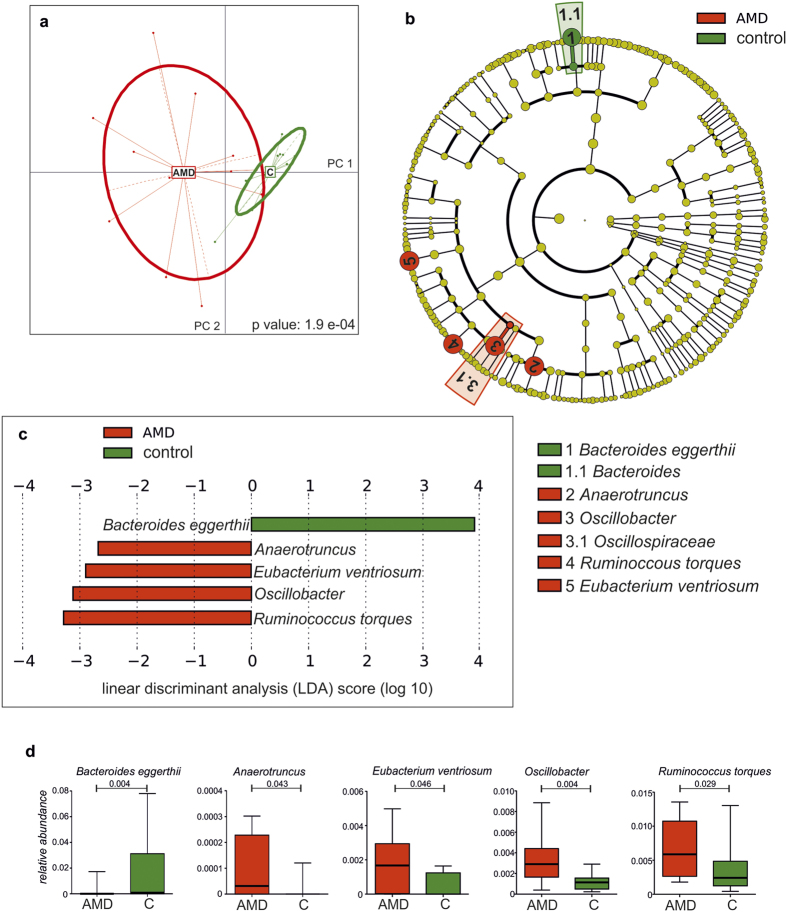
Microbial composition associated with neovascular age-related macular degeneration (AMD). (**a**) Principal component analysis of microbial species abundance using health status as grouping variable. The relation between microbial abundance and health status was assessed using Monte Carlo simulations with 10,000 iterations by which a p value was calculated. (**b,c**) Identification of taxonomic features relevant in neovascular AMD using LefSe. Cladogram for taxonomic representation of significant differences among AMD and controls. The diameter of each circle is proportional to its abundance (**b**). Histogram of the LDA scores for differentially abundant taxonomic features among groups. The threshold on the logarithmic LDA (linear discriminant analysis) score for discriminative features was set to 2.0 (**C**). (**d**) Box plots representing the mean abundance +/−SD of bacterial genera and species associated with AMD (Kruksal-Wallis test, p ≤ 0.05). Red are patients (AMD, n = 12), green are controls (C, n = 11).

The R package ade4^40^ performing PCA was used to provide the global analysis of microbial species abundances between AMD and controls as described in http://www.sthda.com/english/wiki/ade4-and-factoextra-principal-component-analysis-r-software-and-data-mining. PCA was performed with scaled values on relative abundances of microbial species identified by MetaPhlAn (Fig. 1b). A visualization of the individual samples grouped by the classes (case and control) is provided in Fig. 3a. Monte Carlo simulations with 10,000 iterations were performed on the between-groups inertia percentage by which a p value was calculated in Fig. 3a.

To identify taxa with significantly different relative abundances in AMD and controls, relative abundances from the different samples were merged and the linear discriminant analysis effect size (LEfSe) algorithm^41^ was applied with default settings (p < 0.05 based on Kruksal-Wallis test and LDA score ≥ 2).

### Functional profiling

**Figure 4:**
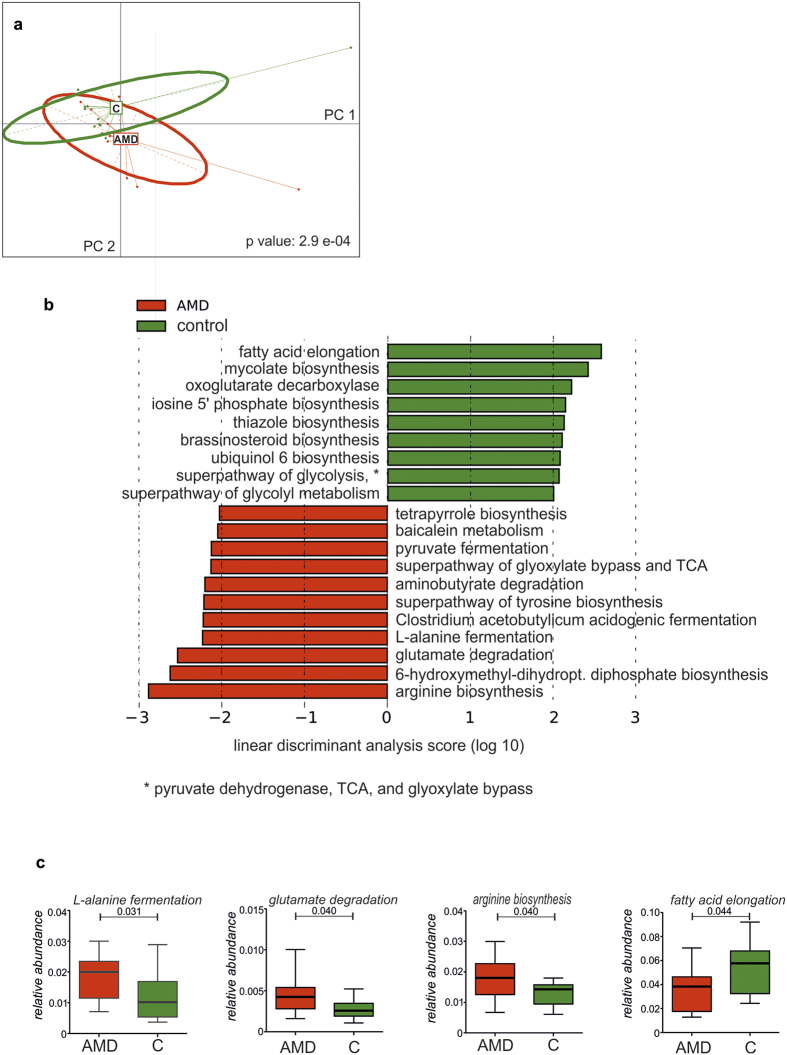
Species-specific microbial pathways associated with neovascular age-related macular degeneration (AMD). (**a**) Principal component analysis of microbial pathway abundance with health status as instrumental variable. The relation between pathway abundance and health status was assessed using Monte Carlo simulations with 10,000 iterations by which a p value was calculated. (**b**) Metabolic pathways that are differentially abundant among subjects with AMD and controls, ranked according to the effect size (LDA score, linear discriminant analysis score). (**c**) Box plots representing the mean abundance +/−SD of pathways associated with AMD (empiric p > 0.05). Red is patients (AMD, n = 12), green is controls (C, n = 11).

To describe the metabolic potential of the identified microbes, the HMP (Human Microbiome Project)^16^ Unified Metabolic Analysis Network (HUMAnN2 v.0.2.1) was used with default settings^17^. HUMAnN investigates the presence/absence and the relative abundance of gene families and pathways in each sample to provide a functional interpretation of the metagenomic sequences. HUMAnN was run for each sample separately using the taxonomic profiles from MetaPhlAn. For nucleotide-level searches, Bowtie2 v.2.2.4 was used to map reads to the functionally annotated pangenome database ChocoPhlAn. All unmapped reads were used for translated searches against the universal protein reference database UniRef50^42^ using Diamond v. 0.7.9^43^. The resulting organism-specific gene hits were assigned to pathways using MinPath v.1.2^44^, finally providing a set of pathways and their relative abundances. To provide an empiric null distribution, 10,000 permutations were performed, randomly assigning the pathways to the two groups, and for each permutation the difference of the means was calculated, providing an empiric p value for each feature (Fig. 4c).

The R package ade4^40^ performing PCAwas used to provide the global analysis of pathway abundance between AMD and controls as described in http://www.sthda.com/english/wiki/ade4-and-factoextra-principal-component-analysis-r-software-and-data-mining. PCA was performed with scaled values on relative abundance of genes and pathways identified by HUMAnN (Fig. 1b). A visualization of the individuals grouped by case and control is provided in Fig. 4a. Monte Carlo simulations with 10,000 iterations were performed on the between-groups inertia percentage by which a p value was calculated in Fig. 4a.

To identify differences between AMD and controls, LEfSe was applied to all pathways that had been found in at least 11 of the 23 samples using default settings. A difference was considered to be statistically significant if p < 0.05 (Kruksal-Wallis test) and LDA score ≥ 2.

## Additional Information

**Accession codes:** Gut microbiome sequencing reads have been deposited in the European Nucleotide Archive under accession number PRJEB13835.

**How to cite this article**: Zinkernagel, M. S. *et al*. Association of the Intestinal Microbiome with the Development of Neovascular Age-Related Macular Degeneration. *Sci. Rep.* **7**, 40826; doi: 10.1038/srep40826 (2017).

**Publisher's note:** Springer Nature remains neutral with regard to jurisdictional claims in published maps and institutional affiliations.

## Supplementary Material

Supplementary Figure S1

## Figures and Tables

**Table 1:** Characteristics of study participants.

Feature	Patients (n = 12)	Controls (n = 11)	P value AMD vs C
Males (n)	8	7	1^Δ^
Age (years)	78.4 ± 7.4	72.5 ± 7.0	0.064^*^
Current smoker (n)	1	0	1^Δ^
Previous smoker (n)	6	5	1^Δ^
BMI (kg/m^2^)	27.5 ± 4.8	25.5 ± 4.6	0.31^*^

AMD, age-related macular degeneration; BMI, body mass index; C, control. Data are mean ± SD, ^Δ^Fisher’s exact t test, *Welch’s t test.
