# Supplementary material for: Association of the Intestinal Microbiome with the Development of Neovascular Age-Related Macular Degeneration
Source: Sci Rep. 2017 Jan 17;7:40826. doi: 10.1038/srep40826 (PMC5240106; doi:10.1038/srep40826)
Supplement: Supplementary Figure S1 [file srep40826-s1.pdf]

Association of the Intestinal Microbiome with the Development of Neovascular Age-Related Macular Degeneration

Martin S. Zinkernagel, MD, PhD, Denise C. Zysset-Burri, PhD, Irene Keller, PhD, Lieselotte E. Berger, MD[1], Alexander B. Leichtle, MD, Carlo R. Largiadèr, PhD, Georg M. Fiedler, MD, and Sebastian Wolf, MD, PhD

Supplementary Figure 1

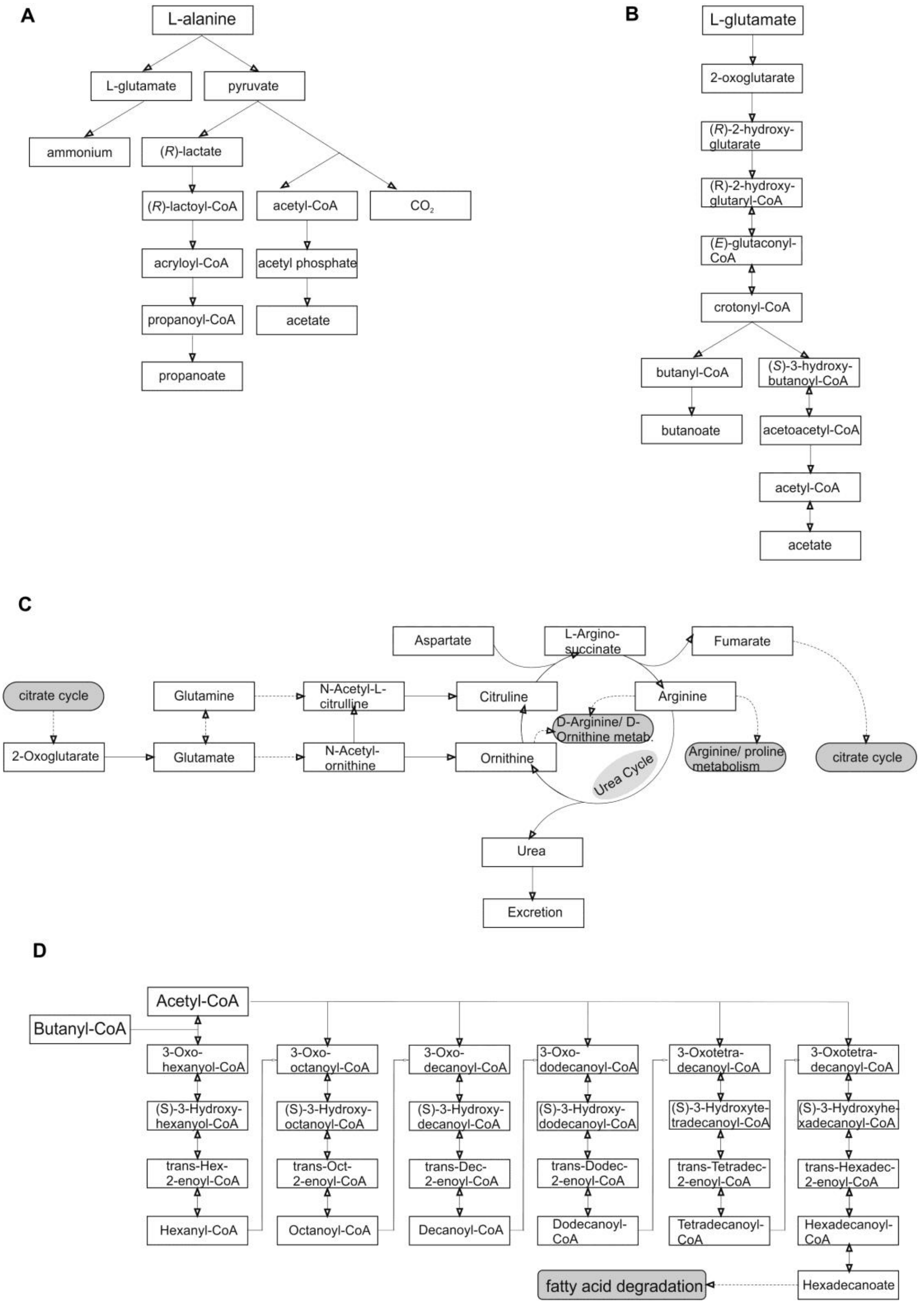

Supplementary Figure S1. Metabolic pathways of the intestinal microbiome associated with age-dependent macular degeneration (AMD). Ilustration of the metabolic pathways that are differentially abundant among subjects with AMD and controls: L-alanine fermentation (A, GO 0019652), glutamate degradation (B, GO 0019671) and arginine biosynthesis (C, GO 0006525) were enriched in patients, whereas fatty acid elongation (D, GO 00062) was reduced in patients with AMD (Kruksal-Wallis test, $p > 0.05$).
